# Medical mortality in an emergency department in Nigeria: the transition is obvious!

**DOI:** 10.4314/ahs.v21i1.23

**Published:** 2021-03

**Authors:** AD Olusegun-Joseph, O Akande, E Otrofanowei, EO Nwoye, OB Olopade, JN Ajuluchukwu

**Affiliations:** 1 Department of Medicine, College of Medicine, University of Lagos, Lagos, Nigeria; 2 Department of Medicine, Lagos University Teaching Hospital, Lagos, Nigeria; 3 Department of Biomedical Engineering, College of Medicine, University of Lagos, Lagos, Nigeria

**Keywords:** Emergency department, duration, mortality

## Abstract

**Introduction:**

The emergency department (ED), a major entry point into the hospital, provides an insight to the type of cases seen, the quality of care and mortality spectrum in a health institution. We aim to identify the spectrum of medical causes of mortality in our ED, the demographic pattern and duration of stay before death.

**Method:**

This is a retrospective study that looked at medical mortality in the ED from January 2004 to December 2009. We obtained data on the demographics and causes of death from the medical records and case notes of the deceased.

**Results:**

A total of 16587 patients were admitted during the period under review, of these 1262 (7.61%) died in the ED. The male to female ratio was 1.58:1.0 [772 males (61.2%), and 489 females (38.8%)]. Mortality was highest among the 20–45 years age range, followed by 46–65 years, >65 years and < 20 years in decreasing frequency [589(46.7%), 421(33.4%), 186 (14.8%) and 66(5.2%) respectively]. The three most common causes of death were stroke 315(25%), HIV related illnesses 126(10.0%), and heart failure 123(9.7%). Most deaths occurred less than 24hours of admission, 550(43.6%), followed by one day (36.0%) and two days (10.8%) post admissions respectively.

**Conclusion:**

The commonest cause of death in the ED was stroke. The burden of death was highest in the younger age group, with most occurring less than 24 hours of admission.

## Introduction

The emergency department is a critical entry point into any hospital. It is a department that provides an insight to the type of cases seen, quality of care available in the institution and serves as a clinical audit.^1^ Mortality rate and spectrum is one of the major health indices that should be evaluated regularly in any institution. ED mortality audit is even more important as it helps to unravel the spectrum of emergency cases in the community, the morbidity burden, the demographic spread, the response and effectiveness of the ED in handling these burdens, as well as the mortality burden.^2^ The profile of cases seen also reflect, to a large extent, the prevailing risk factors and pattern of diseases in the community.^1^–^3^

The profile and pattern of medical conditions in Nigeria is changing, mainly due to rapid urbanization and changing lifestyle.^4^ This change will have a direct impact on the pattern of admission in the medical ED, and by extension the pattern of mortality. With the obvious paradigm shift from communicable to non-communicable diseases in developing countries 5–8, the impact of epidemiological transition and the response by health care providers can be further assessed by reviewing medical mortality in health care facilities, especially the ED^2^,^6^. Two previous studies from our center had looked at acute medical deaths (death within 24 hours) in the emergency, Ajuluchukwu et al^9^, and hypertension-related complications of acute mortality, Mbakwem etal^10^ heralding the impact of epidemiological transition. This present study is looking at all medical deaths in the emergency, from time of presentation beyond the first 24 hours.

We aim to assess the pattern and demographic spread of medical deaths in the ED in our center. Such knowledge will provide an insight to the prevailing health challenges in the larger society and assess our health care system. Also the data can help in policy formulation and allocation of resources for health services that will benefit both patients and physicians 11–13.

## Methodology

This is a retrospective study that looked at medical mortality in the ED from January 2004 to December 2009. We obtained data on the demographics, causes of death and duration of stay before death in the ED from the medical records, and the case notes of the deceased.

Our Adult ED has three major arms: Medical, Surgical/Trauma and Obstetrics and Gynecological arm. Children emergency is in another building, far away from the adult ED. This study is focused on death in the medical arm of the ED. The Medical ED at the time as described in a previous work^9^ had three consultant physicians, one Cardiologist and two Nephrologists. They are supported by a team of senior registrars, registrars and house officers. The patients on arrival are quickly reviewed by a doctor in Medicine who assesses to know the case and then refer to the appropriate division. If it is a medical case the Consultant with the senior registrar starts seeing the patient. In the event of a very busy period, the Consultant starts seeing the critical patients and assigns the less critical patients to the senior registrars to attend to. Upon stabilizing the patients, the medical team on call (comprising of a Consultant Physician, one Senior registrar, one registrar and 2–3 house officers) is informed to continue resuscitation and management while the medical casualty team returns to attend to new patients just arriving. The medical team on call (MTOC) informs the specific specialty for the case and admits to the ward. The patients that need intensive care unit admission are reviewed by the Anesthetist on call.

The Nursing staffs in the ED are made up of a mixture of specialty nurses trained in emergency care management and general nurses.

### Study population

Patients with medical cases that died while on admission in the emergency room during the period of study.

**Inclusion criteria:** Medical cases resulting in death in the adult ED as determined by the triage officers, casualty and specialist doctors in medicine, which were documented in the case notes and entered into the medical mortality record book.

**Exclusion criteria:** 1. Non-medical cases as determined by the triage officers.

2. Cases certified as dead on arrival (DOA) or brought in dead (BID) by the triage officers.

Diagnoses were mainly based on clinical presentations. Diagnosis of stroke was made in patients who had sudden numbness or loss /reduced power in one side of the body, with or without history of associated slurred speech, loss of consciousness, on the background history of hypertension or diabetes mellitus. These patients also had elevated blood pressures on admission. Brain CT scan showing hemorrhage or ischemia in the brain also confirms stroke. Heart failure was diagnosed based on history of progressively worsening dyspnea on exertion, orthopnea and paroxysmal nocturnal dyspnea, tachypnea, third heart sounds with or without Chest X ray that showed upper lobe diversion, Kerley B lines, perihilar fullness. Renal failure was diagnosed based on history of Nocturia, frequency, reduced or no urine production, hiccough, with or without flapping tremor. It is also diagnosed in the presence of elevated creatinine and urea, with or without hyperkalemia.

Results were analyzed using the SPSS version 17 statistical package.

Ethical approval was obtained from the Health Research and Ethics Committee in our institution.

## Result

**Figure 1:**
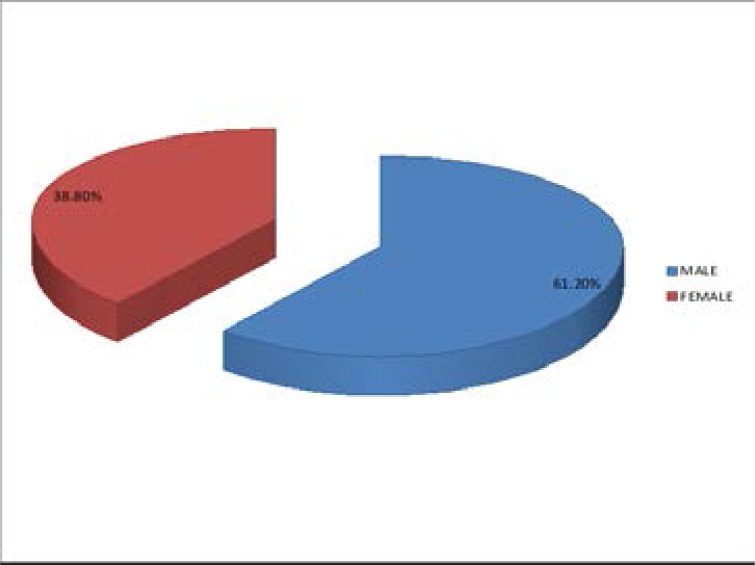
Gender distribution of the deceased

**Figure 2:**
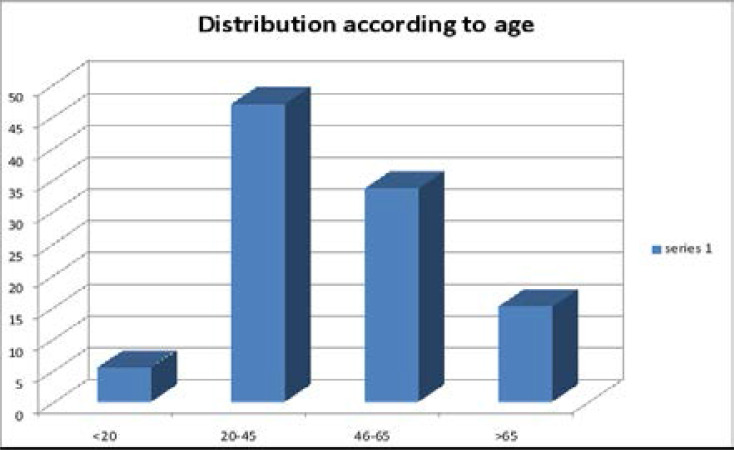
Age distribution 20 years (5.2%); 20–45 years- 589(46.5%); 46–65 years 421(33.4%); >65% – 186 (14.8%).

A total of 16587 patients were admitted during the period under review. Of these, 1262 (7.61%) died in the ED. The male to female ratio was 1.58:1.0 [772 males (61.2%), and 489 females (38.8%)] as shown in Figure 1. Mortality was highest among the 20–45 years age range 589(46.7%), followed by 46–65 years, >65 years and <20 years (18–19 years) in decreasing frequency [421(33.4%), 186 (14.8%) and 66(5.2%) respectively] as shown in figure 2.

**Figure 3:**
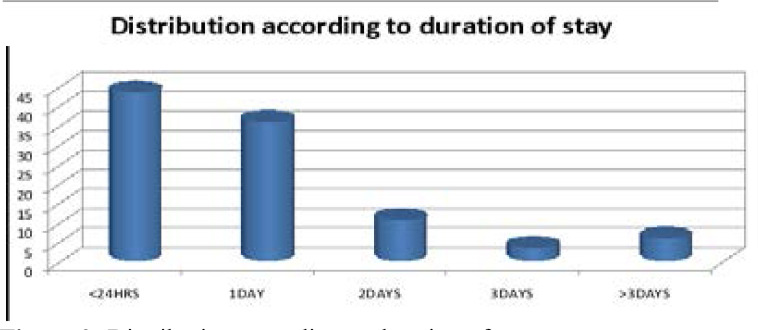
Distribution according to duration of stay <24 hours- 550(43.6%); 1day – 454(36.0%); 2days- 137(10.8%); 3days- 45(3.6%); >3days – 76(6.0%).

The three most common causes of death were stroke 315(25.0%), HIV related 126(10.0%), and heart failure 123(9.7%). This was the pattern each year during the period of study. Details of the causes of death are shown in table 1. Cardiovascular disease accounted for 50.48% (637) of the total death, while infectious disease accounted for 28.05% (354). The remaining 21.47% were from other forms of non-communicable diseases like chronic liver disease, gastrointestinal bleeding etc. Unclear diagnosis and causes of death that were not more than 5 (0.40%) were classified as miscellaneous. Most deaths occurred less than 24hours of admission, 550(43.6%), followed by one day (36.0%) and two days (10.8%) post admissions respectively as shown in figure 3.

**Table 1:** Cause of mortality distribution

Cause of mortality	Frequency	Percentage
Cerebrovascular Disease	315	25.0
Retroviral disease	126	10.0
Heart failure	123	9.7
Hyperglycemic emergencies	120	9.5
Septicaemia	86	6.8
Chronic kidney disease	79	6.3
Sickle cell disease	49	3.9
Tuberculosis	48	3.8
Chronic liver disease	46	3.6
Meningitis/Encephalitis	36	2.9
Tetanus	33	2.6
Gastrointestinal bleeding	30	2.4
Cerebral malaria	18	1.4
Primary liver cell carcinoma	7	0.6
Toxic hepatitis	7	0.6
Miscellanous	139	11.0

**Total**	**1262**	**100.0**

## Discussion

Emergency department mortality audit is one of the major health indices that should be evaluated regularly in any institution as it is a reflection of the spectrum of cases in the community, the morbidity cum mortality burden, and demographic spread. Our institution serves as a referral center to many hospitals within the state and many neighboring states. This study, therefore, possibly reveals, to a large extent, the mortality burden and spectrum across several states in Nigeria.

Mortality rate in our study was 7.61%. This is quite high when compared with other studies. Ekere^1^ reported a mortality rate of 2.2%, Runkewe et al 6.0%^14^, while Onwuchekwa et al revealed a rate of 6.8%^15^. Ekere^1^, Adesunkanmi^5^ and Runkewe^14^ et al reported mortality in the whole emergency department, it would have been more comparable if medical deaths alone were isolated. Even at that, medical emergency deaths were reported to be the highest in their studies, accounting for more than 50% of total mortality. Our finding is linked with late presentation as most patients present when chances of salvaging the situation looked very bleak. In addition, overstretch of facility played a major role, as the period of study was riddled with multiple industrial actions in the secondary care centers and the other tertiary center in the state, making our center the only functional referral facility at such times. Our center, however, also had its own fair share of industrial dispute during the period of study, accounting for the relatively low overall admission during the period under review. This, unfortunately, may also be a de facto contributing factor to the relatively high mortality we recorded, since most patients during dispute periods were initially managed in private hospitals that had no requisite skill and support system to handle such cases; and once the dispute ended, extremely critical, poorly managed patients present in the hospital in droves with very little chance of survival.

Mortality rate locally, including our center, is quite high when compared with similar studies from industrialized countries where mortality rate is as low as <0.30%^16^,^17^. Unlike this countries where paramedics would have started life saving intervention right from home and patients taken to the hospital in adequately equipped ambulance, most of our patients are brought to the hospital in private cars or commercial vehicles, with no form of life saving intervention before arrival. This unfortunate trend is prevalent locally 1,18. The end result is that a number of patients have more complications on arrival with very little chance of survival. Increased paramedics personnel with improved lifesaving pre-hospital management, provision of adequately equipped ambulance for transportations of patients to point of care, stability in the health sector, improved first line management at referral hospitals, early referrals and presentation will help to reduce high mortality in our ED.

Our study revealed a higher mortality in males than females, 1.58:1. Similar findings were reported by other workers^1^,^5^. Ekere et al^1^ and Adesunkanmi et al^5^ both reported a male to female mortality ratio of 1.5:1 in their respective studies. The higher mortality rate in males compared to females appears to follow a perennial, global trend as other researchers both local and foreign observed similar pattern^14^–^23^. Male gender are more likely to die from cardiovascular and many other illnesses due to many risky practices and unhealthy behaviors like smoking, alcohol and such likes that they indulge in^23^–^25^. Other reasons that make the male gender more at risk of death at every given age than the female counterpart include genetic, hormonal and biological differences^26^–^29^. Improving the health and morbidity outcomes of men must receive more attention globally.

A very disturbing finding in our study is that most of the deceased were young, with >50% within 45 years bracket. This grave pattern has been consistently reported by other investigators 1,5,9,14–15. Our finding is, however, at variance with studies from the western world, where most of the dead were much older, ≥ 70 years 16–17,21–22. Our finding is a cause for concern, as this portends wastage of our working population and most productive age group, with resultant decreased productivity, increased number of orphans and fractured societal framework cum support system. This will worsen the poverty level in the society with increased morbidity and mortality, thereby setting up a vicious cycle between poverty, morbidity and mortality^6^,^30^.

Most death in our study occurred less than 24 hours, with majority occurring within 6 hours of admission. This is similar to both local and international studies^1^,^9^,^10^,^20^. Late presentation and poor pre-hospital management are important contributing factors. Furthermore, mismanagement in primary referral center, shortage of trained, experienced emergency medical personnel, coupled with lack of adequate supportive and lifesaving equipment are also contributing factors. The creation of intensive or highly dependent unit linked to the ED may help to reduce the trend and save more lives.

Cardiovascular disease (CVD) accounts for 50.48% (637) of the total death, with stroke being the commonest cause of death. This was consistent throughout the whole period of study. Similar finding was previously reported in our center^9^, other parts of the country as well as high income countries^1^,^5^,^15^–^17^,^20^,^22^. A worthy difference between our finding and studies from industrial countries, however, is that while they have more atherosclerotic complications as the leading cause of death, presenting as Acute Myocardial infarction, occurring in older age group^10^,^16^; we had more cases of vascular complications of hypertension, presenting as stroke, hypertensive heart failure, and chronic kidney disease from hypertensive nephrosclerosis in younger age group^10^,^18^,^31^. CVD is clearly the leading cause of death in our country as it is globally; having long overtaken infectious disease, which by the way is still a force to reckon with. The main reason for this is a massive upsurge in the risk factors in our society^4^,^9^, early onset of disease coupled with late diagnosis^9^,^32^, poor adherence to medications^33^ and rapid progression of disease^32^,^33^. Aggressive education, early diagnosis and management of risk factors will greatly help to reduce this epidemic that is mercilessly consuming our citizens, especially the young ones 6,9,10,33.

Our study also revealed that infectious disease is still a relevant cause of death in our society, accounting for 28.05% of total death, and complicating about 15% of non-infectious causes of death. Retroviral disease associated death was the commonest cause of death in this category, in keeping with other local reports^15^,^18^,^20^. This further amplify the importance of continual awareness campaign, early diagnosis and institution of management with Anti-Retroviral therapy (ART) once diagnosed to reduce morbidity and mortality of this scourge^34^–^36^. It is rather disturbing that Tuberculosis, Cerebral malaria, meningitis and tetanus are still contributing to mortality in our society. These are illnesses that thrive in the presence of poverty, crowded environments with poor level of hygiene^37^–^38^. Furthermore, they are complications of poor management^37^,^39^–^40^. The need to heighten our campaign for cleanliness, adequate housing, primary prevention strategies like vaccination, and early treatment of illnesses cannot be overemphasized.

There are few limitations in our study. This is a retrospective study, and we had issues with incomplete data in some of the records. A number of the patients, especially those that died less than 24 hours following presentation were unable to complete outlined investigations before their demise, making their cause of death largely clinically based. Furthermore, there was no pathologist (autopsy) report to confirm the cause of death. Clinical certification has its own limitation when compared to autopsy and has been shown to be less accurate^41^. However, a previous autopsy study on acute medical death in our center^9^ reported findings that follow the same trend as ours, lending credence to our findings.

## Conclusion

Medical causes of mortality in our ED, as far back as over a decade ago, were a blend of non-communicable and communicable diseases, with cardiovascular diseases accounting for over 50% of cases. This amplifies the impact of epidemiological transition is our society even as at that time. Most patients died within 24 hours of admission, with late presentation being a major challenge. Unfortunately, the young in the prime of their productive years, were most hit! This is a wakeup call for aggressive enlightenment, screening, early diagnosis and management of cardiovascular risk factors on one hand, and improved hygiene, living condition, vaccination, eradication of poverty with prevention and early treatment of infectious diseases on the other hand. The second part of the study will look at mortality pattern from 2010 to 2019, once ethical approval is given, and compare it with this present study.
